# Demonstration of a length limited parametric array

**DOI:** 10.1016/j.apacoust.2019.01.001

**Published:** 2019-05

**Authors:** Elizabeth Skinner, Matthew Groves, Mark K. Hinders

**Affiliations:** William & Mary Applied Science Department, Williamsburg, VA 23187-8795, United States

**Keywords:** Nonlinear sound propagation, Parametric acoustic array, Length-limited sound beam, Parametric loudspeaker, Directional sound beam

## Abstract

We describe a series of measurements to assess the practicality of a length limited parametric array in air. This study shows that the length limited effect is a measurable phenomenon that can be produced using pairs of commercial off the shelf parametric array speakers. We generated the effect using parametric arrays mounted so that two directional audio beams were simultaneously co-propagating through the open air. Parametric arrays work such that after the ultrasound frequencies have attenuated, the remaining audio range acoustic frequency is linear. We used this principle to propagate 2 kHz signals from two parametric array speakers, adjusting the relative phase of the resulting audio-range signals to produce varying amounts of constructive or destructive interference in the resulting linear sound beams. We demonstrated that increasing the overlap of the audible sound beams increased the effectiveness of the length limited phenomenon. We also found that changing the magnitude of the sound projected through one of the speakers did not have significant impact on the length limited effect.

## 1 Introduction

Parametric acoustic array speakers create highly directional beams of audible sound by simultaneously transmitting two ultrasound frequencies [1], [2], [3], [4], [5], [6], [7], [8], [9]. The nonlinearity of air creates both a sum and difference frequency as the overlapping ultrasound beams propagate [10], [11], [12], [13]. Since attenuation is proportional to frequency squared, the yet higher sum frequency and original ultrasound frequencies attenuate very quickly while the low difference frequency continues to propagate through the air with similar directionality to the original ultrasound frequencies.

**Fig. 1:**
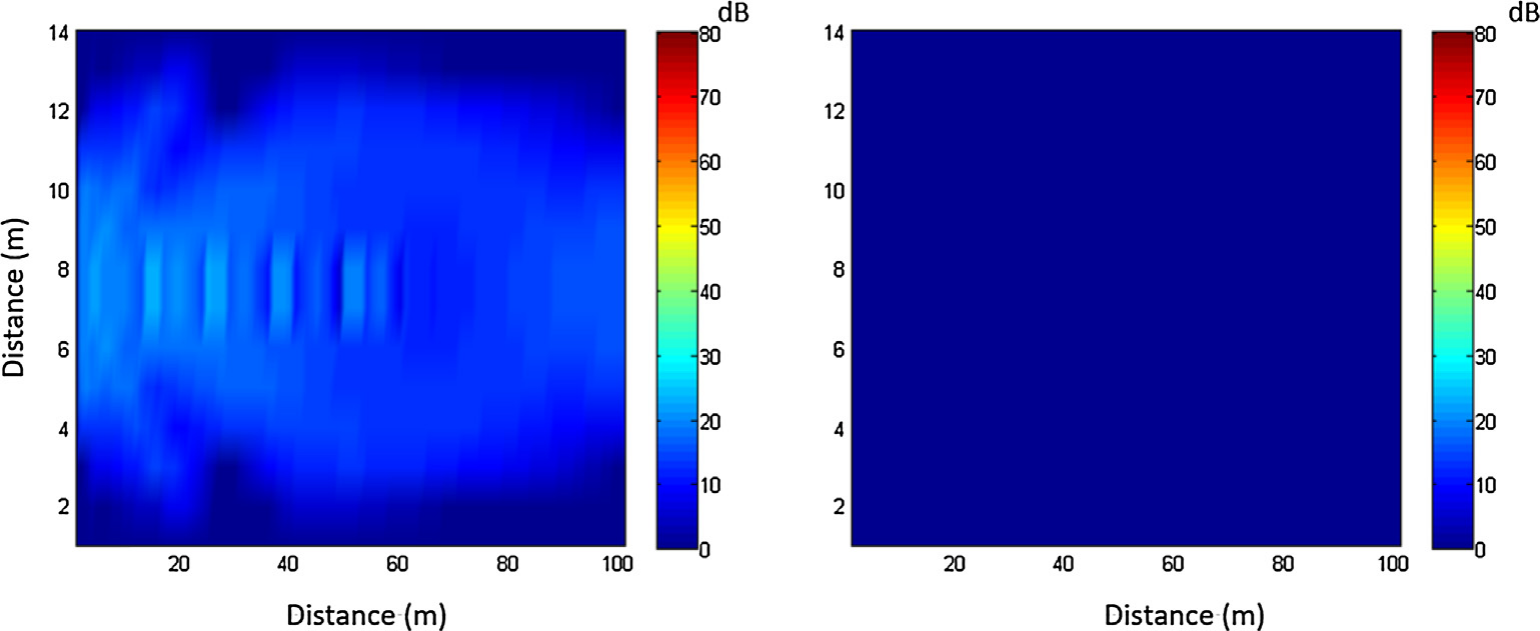
Sound pressure levels of the 5 Hz difference frequency. In the left plot the beam pattern can be seen in the lighter blue, while the right image has no beam pattern because the nonlinearity parameter was set to zero.

Parametric arrays create a narrow beam of audible sound due to the non-linearity of air. Fig. 1 illustrates the role nonlinearity plays in generating the acoustic signal for the parametric array. For this simulation the radius of the emitter aperture is 0.3 m and the primary wave frequencies are 47,500 Hz and 52,500 Hz. These two colormap plots were generated with simulations of the parametric array using the Texas KZK code. In these colormap plots the nonlinearity parameter on and off, respectively. These plots show the sound pressure levels in dB of the 5 Hz difference frequency. The lighter blues represent higher sound pressure levels, while the dark blue represents areas of relative quiet. The plot on the left shows a simulation where the nonlinearity parameter is set to that of air. In this plot the beam pattern of the 5 kHz signal can be seen in the lighter blue. The right image has no 5 kHz beam pattern because the nonlinearity parameter was set to zero, illustrating that it is the nonlinearity of air that makes these directional speakers possible.

There have only been a few parametric array speakers available in the consumer market, including the Holosonics AudioSpotlight [14], the HyperSound HSS300 [15], the Acouspade [16], the Sennheiser Audiobeam [17], and the Soundlazer, a KickStarter project [18]. Most parametric array speakers are very expensive due to the significant amount of signal processing required for producing quality audio [7] and small-volume production. Parametric array speakers seem to have limited utility in smaller indoor installations because the directional sound reflects off of any hard surface, causing the sound to then be audible throughout the room, which often defeats the purpose of directional sound. It is also difficult to produce a wide range of audible frequencies using a parametric array because the non-linear effect varies with frequency. Hence, using ultrasound to create audible signals does not reproduce the lower frequencies well, which has caused some to add a subwoofer to make the audio quality better [19].

**Fig. 2:**
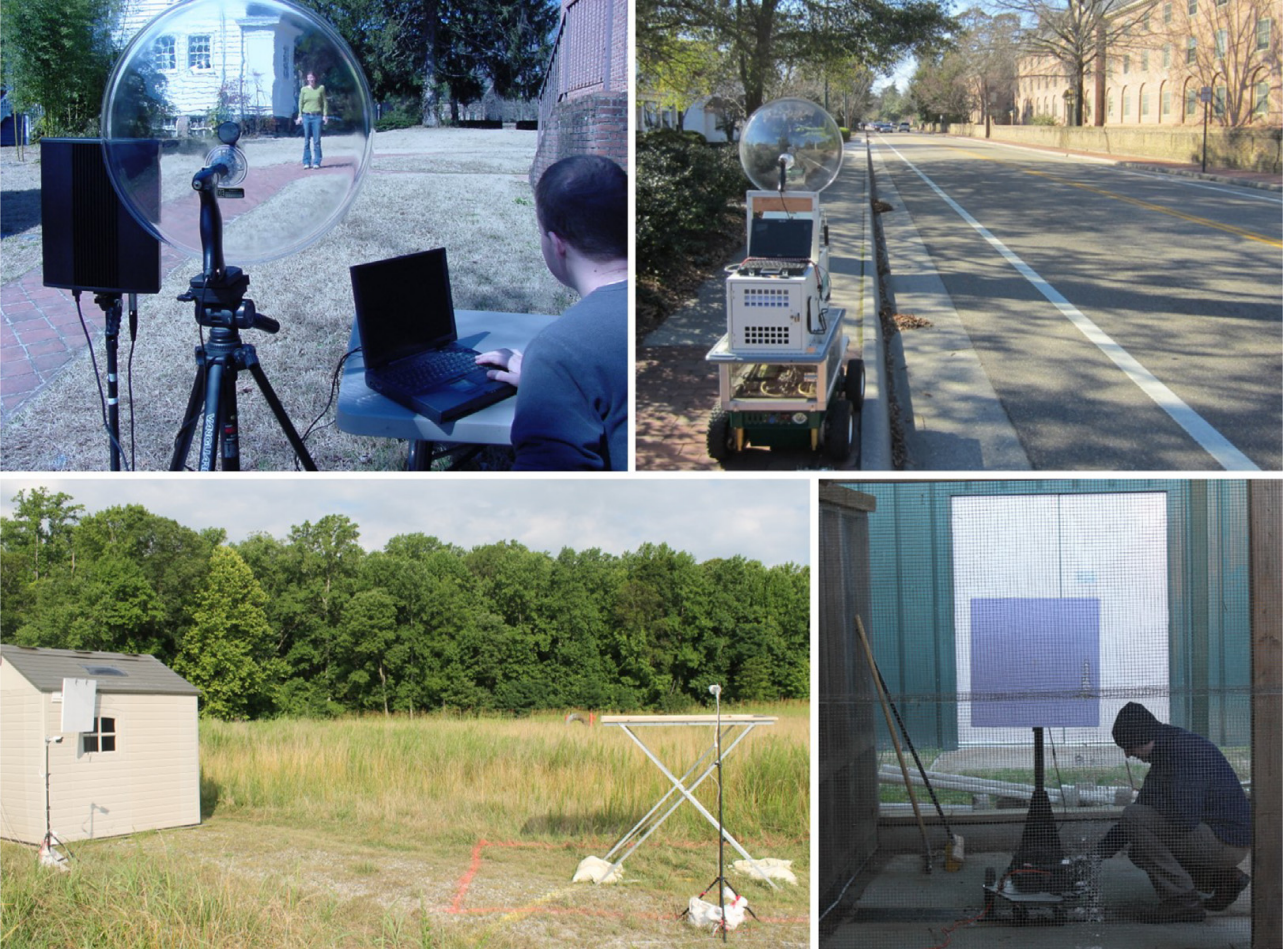
This figure shows some of the projects where we have used parametric arrays. The top left image shows using parametric arrays for concealed weapons detection, where the Sennheiser parametric array beam is reflecting off of the person in the photo and then being picked up with a parabolic microphone. The top right shows the experimental setup for echolocation using the Sennheiser parametric array mounted on the front of a mobile robot rMary. The bottom two images are from aviary and field tests that we performed demonstrating the ability of Sonic Nets to deter birds from feeding location using the 24i AudioSpotlight speaker from Holosonics.

We have been using parametric arrays for many years for a variety of applications ranging from mobile robotic navigation [20], [21], [22], [23], [24], [25] to stand-off concealed weapons detection [26], [27], [28], [29], [30], [31], [32], [33], [34] to benign bird deterrence [36], [37], [38], [39], [40]. Fig. 2 shows some of the research projects for which we have used parametric arrays. For concealed weapons detection, the reflections from the Sennheiser Audiobeam were analyzed to determine if the person was carrying weapons under their clothes. The concealed weapons work showed that the audible beam created by the two ultrasound frequencies could be focused using a shaped parametric array [32]. This allowed for an audible beam that could be pointed at a person’s torso without that person being able to hear the sound [35]. Fig. 2 also shows an experimental apparatus for echolocation using the Sennheiser Audiobeam mounted on the front of a mobile robot rMary [25]. A chirp signal generated by the parametric array is backscattered from vehicles traveling on the road and used to classify the vehicles coming from as far away as 50 m. The other two images in Fig. 2 are from field and aviary testing of Sonic Nets [40] to deter birds from feeding locations [38]. We used the parametric array to cover one localized feeding area with a colored noise signal that overlaps the audible frequency spectra of communication of pest birds, which causes birds to disperse. For all of these applications, the ability to control where the sound beam goes is the key enabling technology.

Parametric arrays allow a compact device to generate narrow beams of audio-range sound, but there are usually confounding issues caused by acoustic scattering from objects and structures distal to the target region. A controllable finite-length sound beam would be a significant improvement for many applications. Recent work has included self-silencing, i.e. length-limiting parametric arrays [41], [42], [43], steerable parametric arrays for noise cancellation [44], [45], [46], [47], directional warning systems for vehicles [48], multiple beams in different directions from a single parametric arrays [49], vertical sound control [50], and omni-directional sound from curved parametric arrays [51].

In 2012 Nomura et al. showed the theoretical existence of a length limited parametric array beam [41] where the beam length can be changed by simply adjusting the relative phase of the difference frequency waves. By transmitting four ultrasound frequencies, instead of two, they could simulate a parametric array beam with a defined end point. Using a finite difference time domain simulation approach they showed that the beam could be truncated and that the resulting beam was yet narrower than the either of the original beams.

In addition to showing the possibility of the length limited effect, this group also showed that the effect could be measured [42]. Measurements were taken when the four ultrasound frequencies were in phase and again when two of the ultrasound frequencies were 180 degrees out of phase. The length limited effect was also measured using eight transducers aligned to produce the four ultrasound frequencies [43]. At 4 m away from the transducers they found that the magnitude of the audible signal was 23 times lower than that of the maximum. Another study showed the length limited effect using two rings of transducers installed at one end of a 6 inch PVC pipe and the amplitude of the audible sound was measured along the length of the pipe [46]. These important results demonstrated that the length limited effect was possible in a laboratory setting.

To evaluate if the length limited effect could be practical on a larger scale we used commercially available parametric array speakers to test the length limited parametric array concept. This experiment was designed to see qualitatively if the length limited effect could be seen using commercially available parametric array speakers. Our goal was to demonstrate the length limited effect in a real-world situation in a large enough open area for the parametric array to propagate without reflecting off of distal walls, obstacles, etc. Our results show that the length limited effect is controllable and can be generated through modulation of the difference frequency. And that the length limited effect could be replicated without the precise control of the signals creating the ultrasound frequencies.

## 2 Methods and materials

**Fig. 3:**
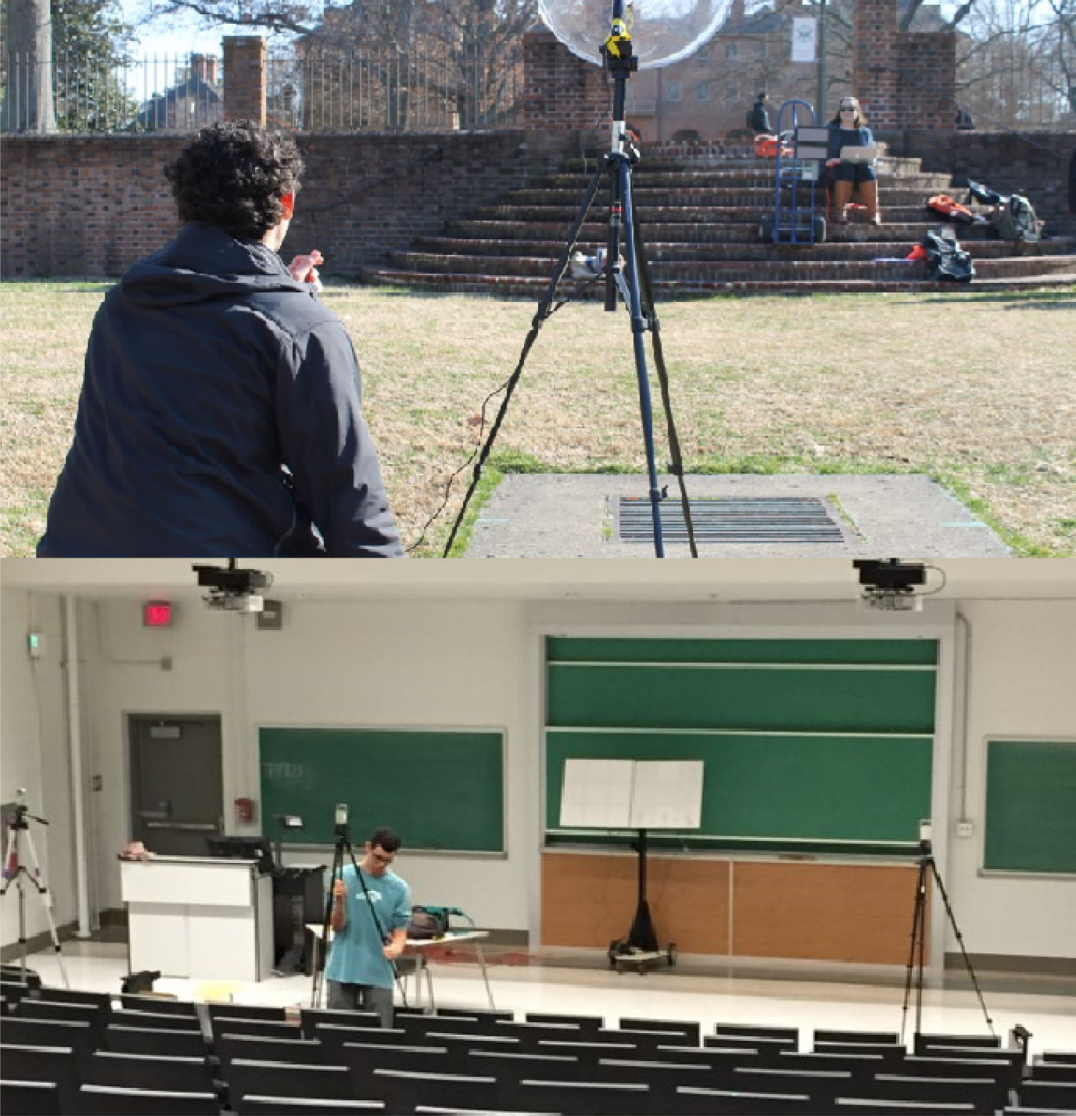
Initial setup for measuring the length limited effect on the Sunken Gardens at William & Mary is shown in the top photo. Here we had two HyperSound parametric array speakers mounted on a cart. Using a parabolic dish microphone we measured the 2 kHz difference-frequency signal from the parametric arrays. We found that there was an audible reduction in volume as we changed the phase of one speaker. The set up for indoor measurements is shown in the bottom picture. We mounted two parametric array speakers in parallel at the front of the lecture hall, pointed towards the back wall, and recorded the acoustic beam with three audio recorders sequentially at increasing distances from the speakers.

Parametric arrays work such that after the ultrasound frequencies have attenuated, the remaining audio range acoustic frequency is linear and so the length limited effect is generated through interference between the difference-frequency sound beams propagating simultaneously. We took advantage of the signal processing in the commercial amplifiers of parametric array speakers to propagate a 2 kHz signal from parametric array speakers pairs. Using Matlab [52] we created sound files containing a 2 kHz tone and then used Audacity [53] to play the 2 kHz tones from two parametric array speakers simultaneously. We held the phase of one speaker constant while incrementally changing the relative phase of the other speaker. Fig. 3 shows the initial setup for measuring the length limited effect on the Sunken Gardens at William and Mary. Here we had two HyperSound parametric array speakers mounted side-by-side on a handtruck. Using a parabolic dish microphone, we measured the 2 kHz difference frequency generated by the parametric array effect. We found that there was an audible reduction in volume as we changed the phase of the 2 kHz tone on one speaker, although construction adjacent to the Sunken Gardens included jackhammering so the measurements were not repeatable. We next moved indoors to a large lecture hall, with high ceilings and sound absorbing panels on the back and sides walls, where it would be quiet enough to perform systematic measurements. We mounted two parametric array speakers side-by-side at the front of the room about 1/2 meter away from the wall, and recorded the acoustic beam with audio recorders every 2 m distant, starting about 6 m from the speakers, both on axis and on either side laterally about 2 m from center. The room is about 12 m in width and more than 18 m deep, which minimized unwanted reflections.

For these measurements we compared the length limited effect generated from two different commercially available parametric array devices, the 24i AudioSpotlight [14] from Holosonics and the HSS300 [15] from Hypersound. By taking advantage of the internal signal processing in the amplifiers of the speakers we were able to show the length limited effect using phase modulations of the audible signal. This in turn made it so that we do not know the detailed information of the modulation and driving signals. For each of these speakers we took multiple measurements of the acoustic beam generated as we varied the phase of one of the speakers. For both brands the speakers were mounted to minimize the between the speakers when positioned in line. This resulted in an approximately 1 cm gap between the speakers, which was increased slightly when the speakers were canted in but never exceeded 10 cm. Each measurement consisted of 20 one minute segments, where the phase of one speaker was increased by 0.1*π* radians each segment. This was repeated 6 times to get the recording at each position in the room. The recordings were taken using Zoom H2 audio recorders^1^ mounted on tripods. These recorders have a flat frequency response from 100 Hz to 10 kHz and a sampling rate of 44,100 Hz.^2^ Each measurement was taken using the same audio recorders, in the same lecture hall, and at similar times in the day. We also angled the speakers up slightly so that the beam was interacting with as few obstacles as possible, reducing the reflections recorded by the microphones. Repeating this set up for each variation in speakers, gain of each speaker, or cant angle of the speakers allows us to compare the amplitude of the measurements directly. For these measurements we used the program Audacity [53] to play the 2 kHz tones generated in Matlab [52] on both speakers simultaneously.

## 3 Results

**Fig. 4:**
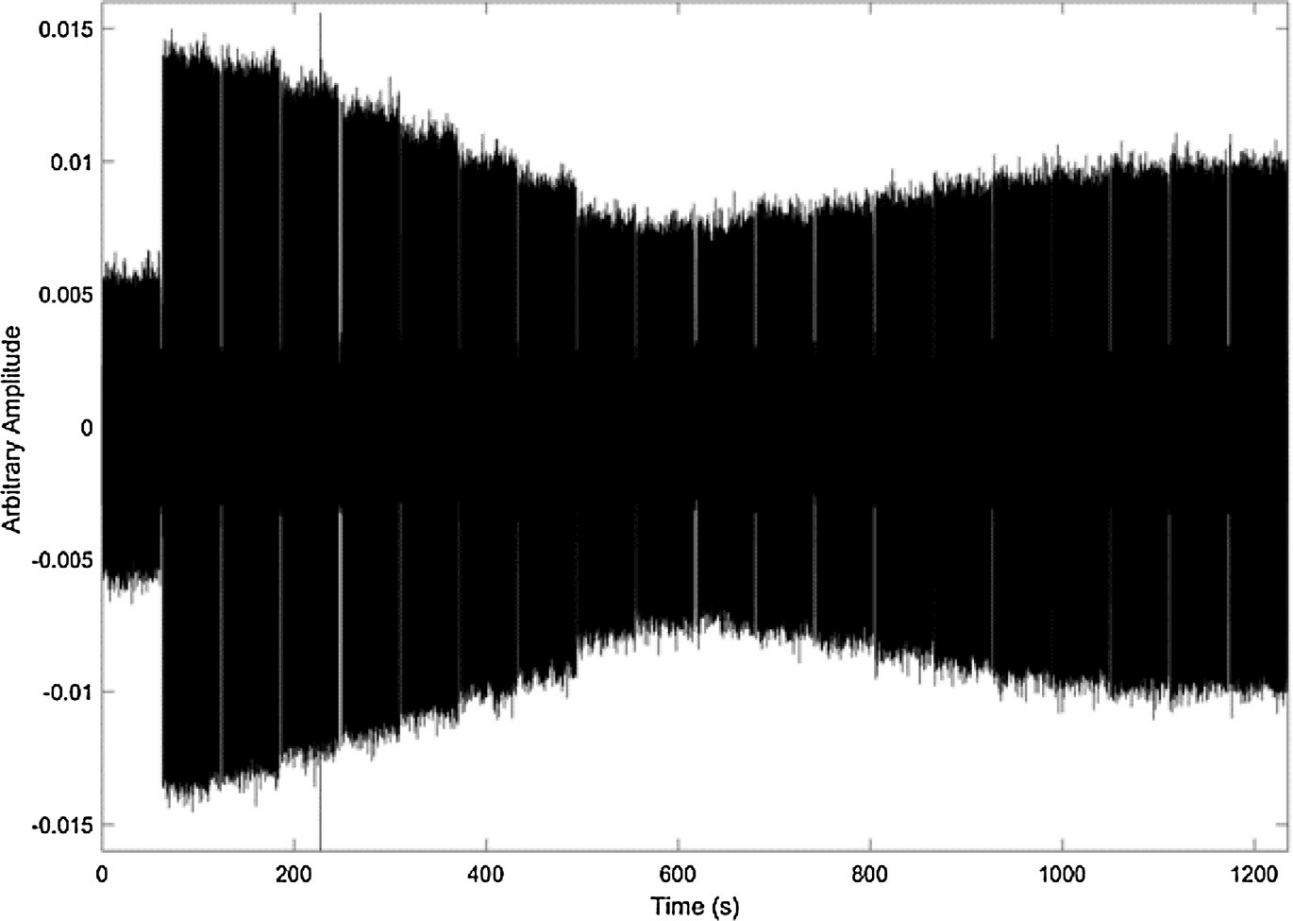
Time trace of recording from about 6 m away from the AudioSpotlight speakers which were positioned in line with one another and with their volume at the same level. We played 1 min of the sine wave and recorded the beam that was produced by the speakers. We then increased the phase of one speaker by 0.1*π* radians and played another minute of those sine waves. This is visible in the plot by the slight stair-step reduction in amplitude of the signal. Each step represents a change in phase of the sine wave. We can see qualitatively that there is a reduction of volume as the speakers are played out of phase.

Fig. 4 shows the recordings from about 6 m away from the speakers. Here the AudioSpotlight speakers were positioned in line with one another and their volume was at the same level for each speaker. For these measurements we played 1 min of the sine waves and recorded the beam produced by the speakers. We then sequentially increased the phase of one speaker by 0.1*π* radians and played another minute of those sine waves. This is visible in the plot by the stair-step reduction in amplitude of the signal. Each step represents a change in phase of the sine wave. This shows qualitatively that there is a reduction of volume as the speakers are played out of phase.

**Fig. 5:**
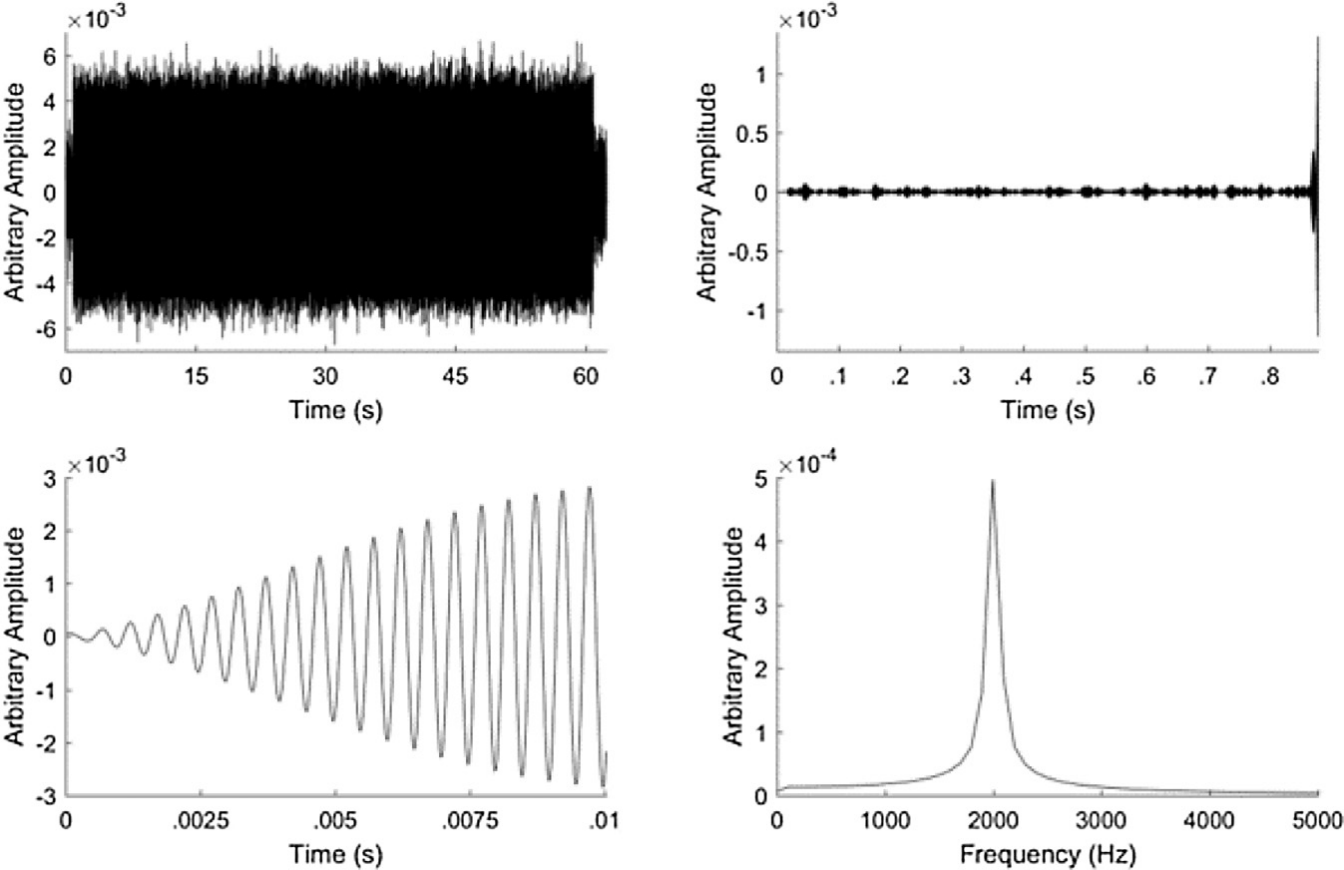
From the filtered signal we segment the files to only contain the 1 min of data for each phase (top left). Then we identify where the 2 kHz tone starts in the recording (top right). We clip the audio to 01 s after the tone starts (bottom left). This ensures that we get the beginning tone without reflections from the back wall of the lecture hall. Then we perform a Fast Fourier Transform (bottom right) and record the magnitude of the peak at 2 kHz.

Fig. 5 shows how we processed the measurements to produce quantitative results. First the recorded signal was filtered to contain only the 2 kHz signal of interest. This removed most of the background noise from the measurements. Next we segmented the files to only contain 1 min of data for each phase. Then we identified where the 2 kHz tone started in the recording and clipped the audio to.01 s after that tone started. This ensures that we get the beginning tone without reflections from the back wall of the lecture hall. Then we performed a Fast Fourier Transform and recorded the magnitude of the peak at 2 kHz.

**Fig. 6:**
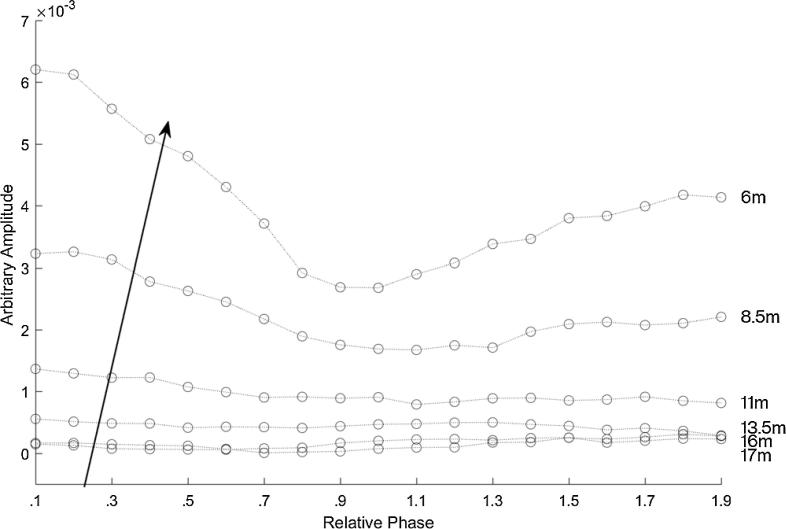
The magnitude of the frequency content at 2 kHz for each recording for the AudioSpotlight 24i speakers positioned in line with each other so that their beams were parallel. Each curve represents a measurement location, with the microphones every two m from the parametric arrays, ordered as indicated by the arrow. The top curve is the position closest to the front, about 6 m away from the speakers, and each circle represents the relative phase in increments of 0.1*π* radians from 0.1*π* to 1.9*π* radians.

Fig. 6 shows the magnitude of the frequency content at 2 kHz for each recording for the AudioSpotlight 24i speakers positioned in line with each other. Each curve represents a measurement location, with the circles indicating changing relative phase of the input sinusoid in increments of by 0.1*π* radians. The top curve is the recording taken about 6 m away from the speakers. The arrow indicates the distance from the speaker where the point is closest to speakers. Here it can clearly be seen that the length limited effect is most apparent in the measurement taken closest to the speakers. The effect is less evident the further away the recorders were from the speakers.

After taking measurements with the AudioSpotlight speakers aligned in parallel and with the audio gain on each speaker the same, we varied the gain between the speakers. The gain levels were set using the gain slider in Audacity. We did not measure if the change in the output in Audacity resulted in an exact 10 dB drop in amplitude from the speaker playing the sound. We were instead interested in how changing the amplitude of one speaker would affect the length limited effect. This was based on discussion in Namora et al. [41] where they simulated the effect with a larger amplitude in one of the parametric array sources. We were concerned that degradation after usage during extended Sonic Nets field tests might have an effect on the volume the parametric arrays were producing. One of the AudioSpotlight speakers had significantly more use and may have been somewhat degraded due to being out in the elements for an extended period. We wanted to ensure that the length limited effect was not due to some anomaly from the speakers not being identical.

**Fig. 7:**
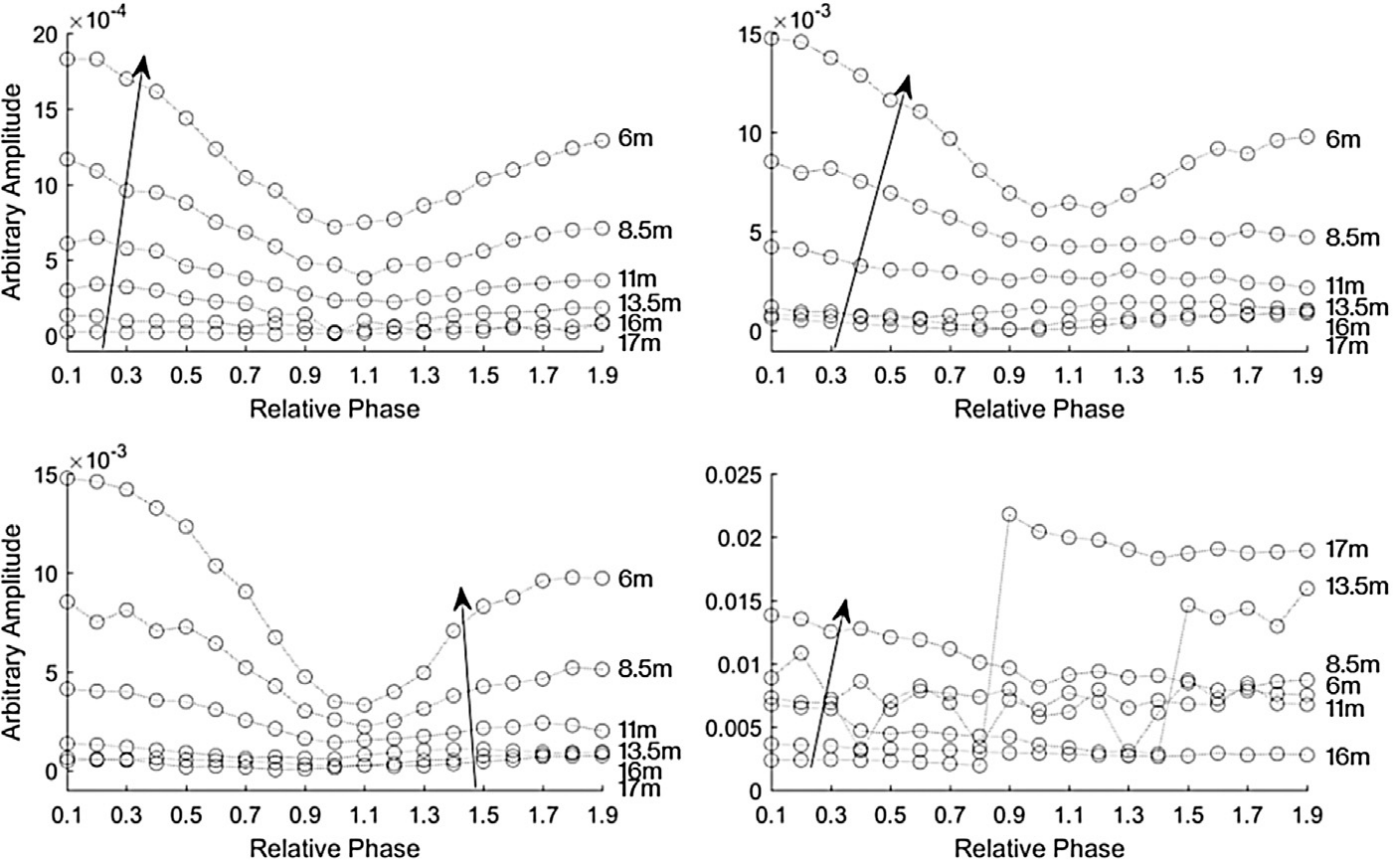
The magnitude of the recorded 2 kHz signal versus phase difference at various distances for the AudioSpotlight 24i speakers positioned in line with each other. The top plots show the measurements with a gain offset of 10 dB. The bottom plots show the measurements with a gain offset of 20 dB. The plots on the right are when speaker one had the lower gain and the left side is when speaker two had the lower gain.

Fig. 7 shows the magnitude of the frequency content at 2 kHz for each recording for the AudioSpotlight 24i speakers aligned in parallel with each other. The top plots show when one speaker had a gain of −10 dB and the other speaker had a gain set at 0 dB. The plot on the right side shows when speaker one had the gain set at −10 dB and in the left plot speaker two had the gain set at −10 dB. The length limited effect is still apparent. We then varied the gain more systematically. The bottom plots in Fig. 7 show the magnitude of the frequency content at 2 kHz of each recording for the AudioSpotlight 24i speakers positioned in line with each other, when one speaker had a gain of −20 dB and the other speaker had a gain set at 0. The plot on the right side shows when speaker one had the gain set at −20 dB and in the left plot speaker two had a gain of −20 dB. Again the length limited effect can be seen in the plot on the left.

**Fig. 8:**
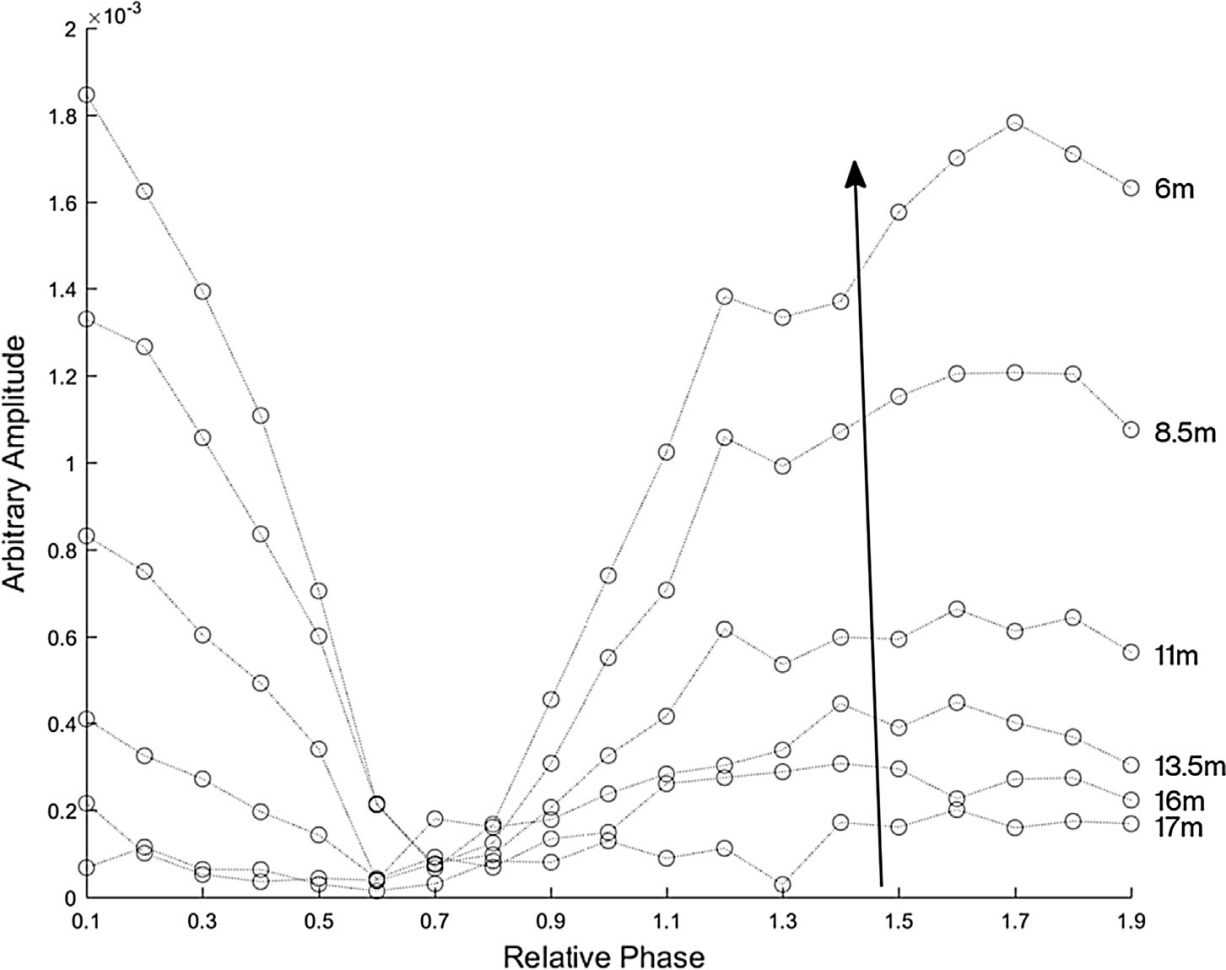
The magnitude of the frequency content at 2 kHz for the AudioSpotlight 24i speakers canted in at 4 degrees to increase the overlap volume of the two beams. Here there is a very dramatic reduction and magnitude between 0.6*π* and 1.0*π* radians phase difference, and the length-limited effect is more pronounced further way from the speakers, e.g. at 10 m away which is the third curve down from the top. Each curve represents a measurement location, with the microphones every two meters from the parametric arrays, ordered as indicated by the arrow. The top curve is the position closest to the front, about 6 m away from the speakers, and each circle represents the relative phase in increments of 0.1*π* radians from 0.1*π* to 1.9*π* radians.

We then repeated all of these measurements with the AudioSpotlight speakers canted inwards at 4 degrees. The effective aperture of the AudioSpotlight speaker is 24 in by 24 in, which creates a quite narrow beam of sound in the acoustic range. Holosonics advertises a beam width of approximately 2 m at 4 m from the speakers [14]. By canting the speakers slightly in, we increase the interaction of the beams and thus increase the length limited effect. Fig. 8 shows the magnitude of the frequency content at 2 kHz of each recording for the AudioSpotlight 24i speakers canted in at 4 degrees, with the amplitude of both speakers set the same. Here there is a rather dramatic reduction in magnitude between 0.6*π* and 1.0*π* radians phase difference. Also the length limited effect is more pronounced further way from the speakers. This is seen at 11 m away (the third line down) when there is still a significant dramatic drop off in the magnitude of the signal.

**Fig. 9:**
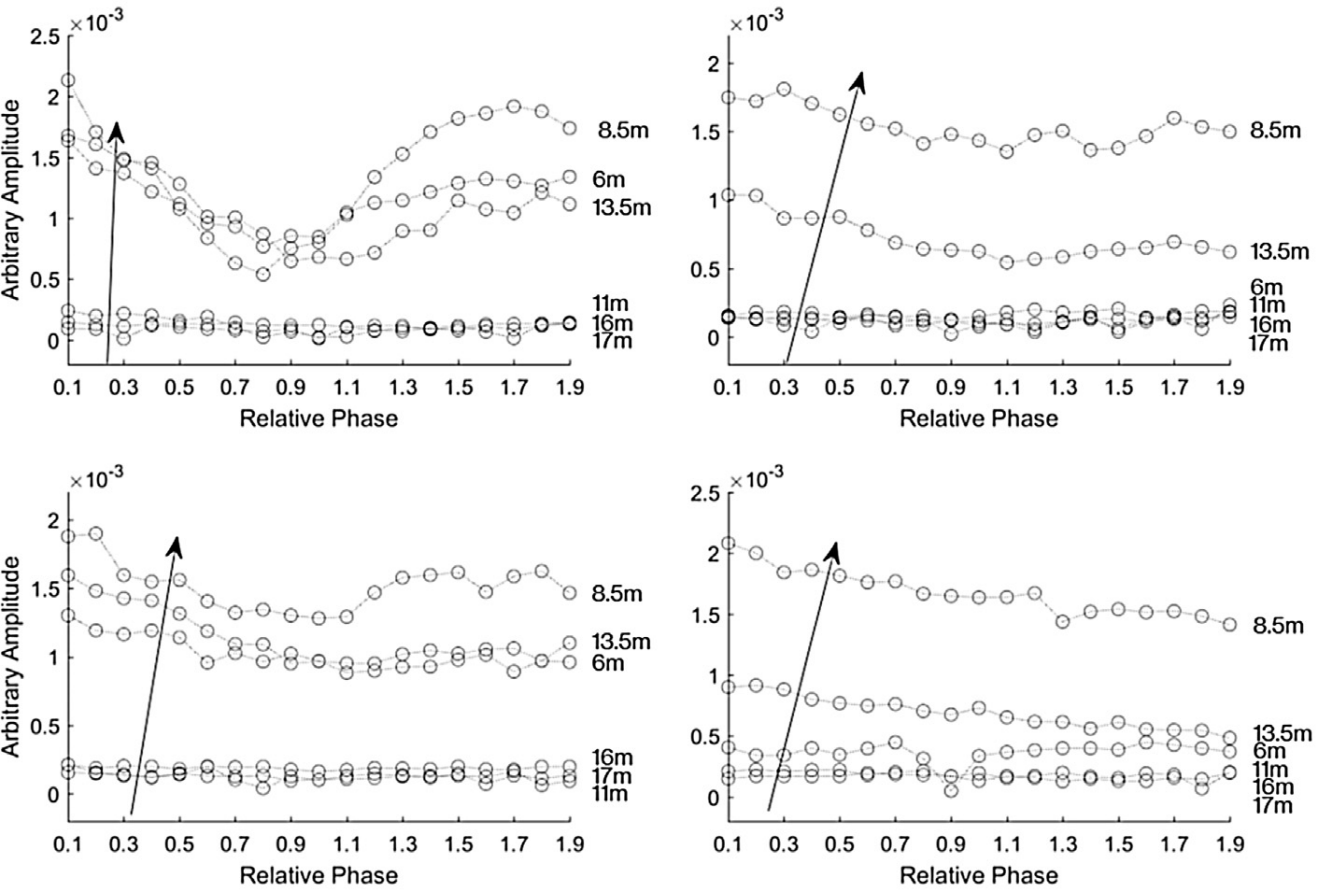
The magnitude of the recorded 2 kHz signal versus phase difference at various distances for the AudioSpotlight 24i speakers canted in at 4 degrees. The top plots show the measurements with a gain offset of 10 dB. The bottom plots show the measurements with a gain offset of 20 dB. The plots on the right are when speaker one had the lower gain and the left side is when speaker two had the lower gain.

Fig. 9 shows the magnitudes at 2 kHz with the speakers canted in at 4 degrees. The top row shows the magnitudes when one speaker had a gain of −10 dB and the other speaker had a gain set at 0 dB. The plot on the right side shows when speaker one had the gain set at −10 dB and in the left plot speaker two had the gain set at −10 dB. The length limited effect can be seen in the 2 measurements closest to the speakers on the left plot, but it is not as dramatic as when the speakers were at the same gain levels. The length limited effect is not apparent in the right plot. The bottom row of Fig. 9 shows the magnitude of the 2 kHz signal from AudioSpotlight speakers canted in at 4 degrees when one speaker had a gain of −20 dB and the other speaker had a gain set at 0 dB. The plot on the right side shows when speaker one had the gain set at −20 dB and in the left plot speaker two had the gain set at −20 dB. Here there is no apparent length limited effect. We collected a similar set of data with the AudioSpotlights canted inward at 10 degrees, but the beam overlap wasn’t sufficient to give a measurable length-limited effect.

The measurements from the AudioSpotlight speakers show that we can produce the length-limited effect using commercially available parametric array speakers while taking advantage of the internal signal processing to create the audible tone. We see in the plots generated from the AudioSpotlight measurements that the data points from the larger relative phases are generally at a lower amplitude than those that should correspond to the same phase difference. For example one would expect that the amplitude of the signal at.1*π* radians offset to be similar to that of the signal at 1.9*π* radians offset. We believe that this is due to the speakers we were using. One of the speakers had been used outdoors for an extended period and the internal elements of that speaker had degraded due to this use. When taking these measurements, it is likely that the amplitude of this speaker dropped slightly as the recordings were taken. Resulting in a slightly lower amplitude overall near the end of the recordings. Even with this slight reduction in amplitude, a significant reduction in amplitude can be see when the offset was between.7*π* and 1.1*π* radians. This is especially true when the AudioSpotlight speakers were canted into increase the beam interaction.

Next we performed similar measurements using the HyperSound [15] speakers. These parametric array speakers have an effective aperture of about 6 inches by 12 inches. For the length limited measurements we mounted them with the longer sides vertically, which should increase the beam interaction by having a smaller effective aperture in the horizontal plane. We expected that by increasing the beam interaction we would increase the length limited effect. Again the speakers were positioned at the front of the lecture hall and the recording devices were positioned throughout the lecture hall. These speakers were nominally identical to each other and fresh out of the box, so we were less concerned about volume variations in the individual speakers.

**Fig. 10:**
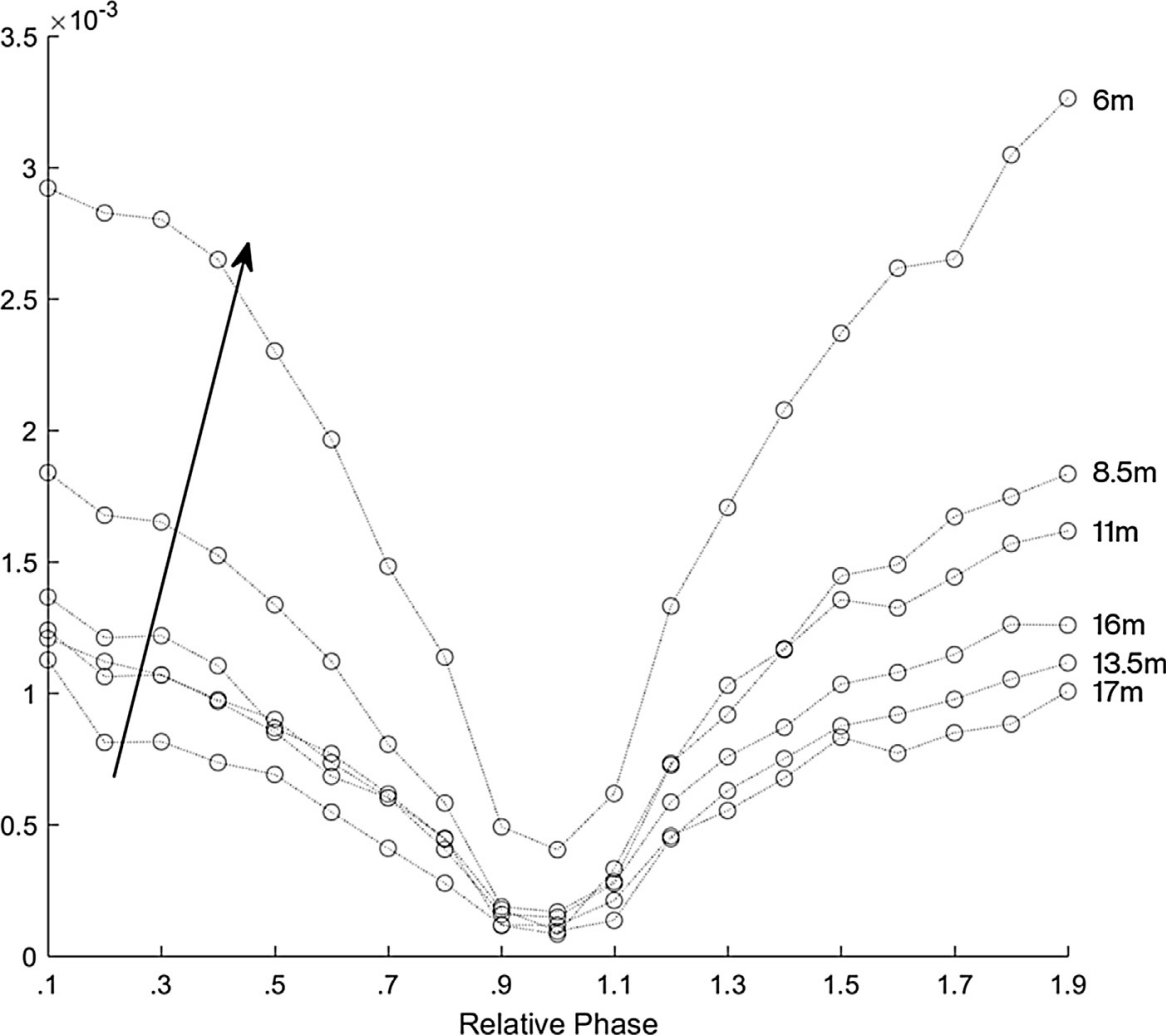
The magnitude of the frequency content at 2 kHz for the HyperSound speakers positioned in line with each other. The reduction in magnitude of the signal is significant at 0.9*π* through 1.1*π* radians out of phase. Throughout the measurement locations, including those close to the back of the classroom, there is a reduction in amplitude as the phase offset gets closer to 1*π*. Each curve represents a measurement location, with the microphones every two meters from the parametric arrays, ordered as indicated by the arrow. The top curve is the position closest to the front, about 6 m away from the speakers, and each circle represents the relative phase in increments of 0.1*π* radians from 0.1*π* to 1.9*π* radians.

Fig. 10 shows the magnitude of the frequency content at 2 kHz for each recording with the HyperSound speakers positioned in line with each other, and with the gain of the speakers set equal to each other. The reduction in magnitude of the signal is significant at 0.9*π* through 1.1*π* radians relative phase. Throughout the measurement locations, including those close to the back of the classroom, there is a reduction in amplitude as the phase offset gets closer to *π* radians. This suggests that having more beam interaction does create a more noticeable length limited effect.

**Fig. 11:**
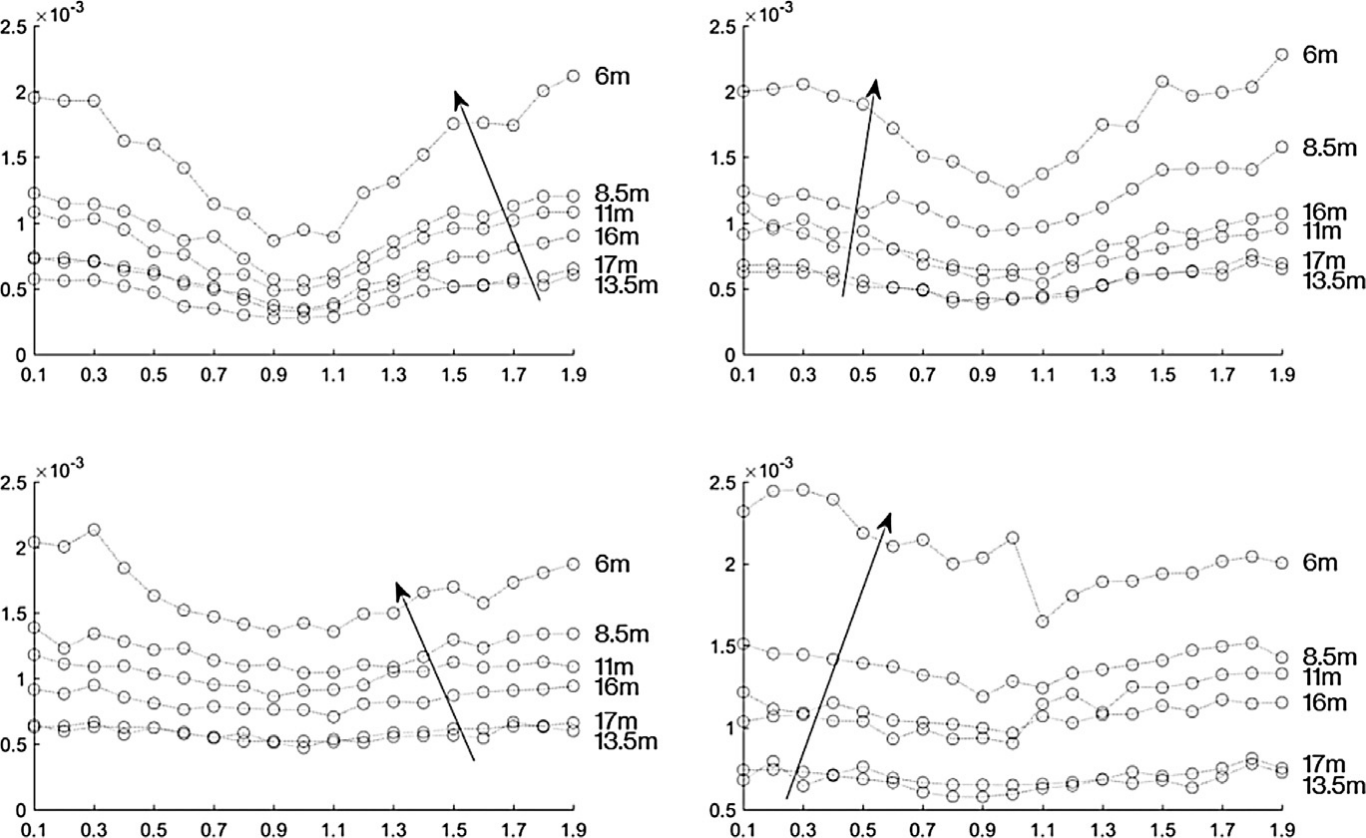
The magnitude of the recorded 2 kHz signal versus phase difference at various distances for the HyperSound speakers positioned in line with each other. The top plots show the measurements with a gain offset of 10 dB. The bottom plots show the measurements with a gain offset of 20 dB. The plots on the right are when speaker one had the lower gain and the left side is when speaker two had the lower gain.

Fig. 11 shows the magnitude of the frequency content at 2 kHz for each recording with the HyperSound speakers positioned in line with each other. THe top plots show when one speaker had a gain of −10 dB and the other speaker had a gain set at 0. The plot on the right side shows when speaker one had the gain set at −10 dB and in the left plot speaker two had the gain set at −10 dB. In these plots the length limited effect is apparent, especially in the measurement taken closest to the speakers. The bottom plots in Fig. 11 show when one speaker had a gain of −20 dB and the other speaker had a gain set at 0. The plot on the right side shows when speaker one had the gain set at −20 dB and in the left plot speaker two had the gain set at −20 dB. It can be seen that having the gain of one speaker at −20 dB had no significant length limited effect in comparison to when the speakers were at the same volume.

**Fig. 12:**
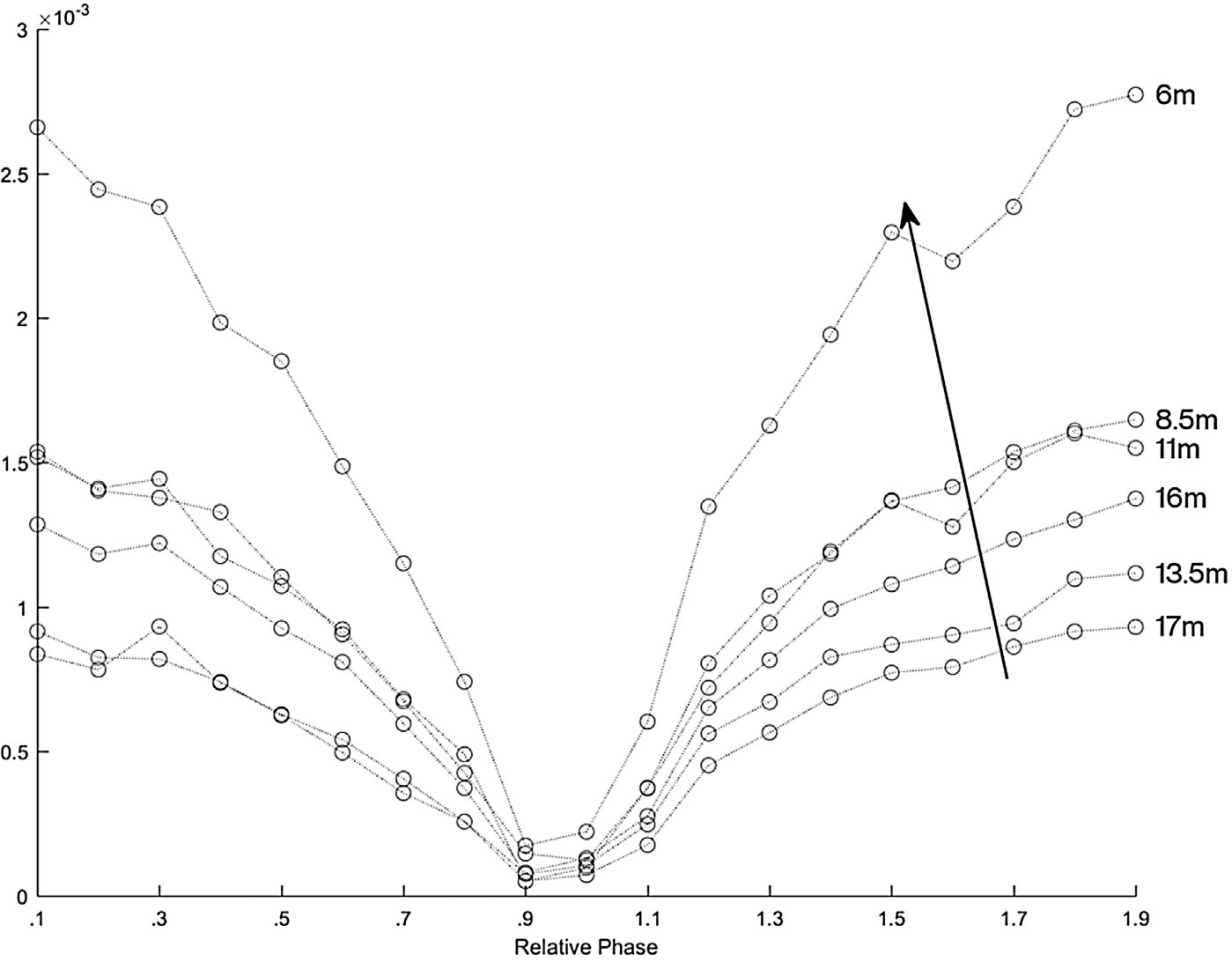
The magnitude of the frequency content at 2 kHz for the HyperSound speakers canted in at 4 degrees, which increases the beam interaction and gives a more prominent length limited effect. Each curve represents a measurement location, with the microphones every two meters from the parametric arrays, ordered as indicated by the arrow. The top curve is the position closest to the front, about 6 m away from the speakers, and each circle represents the relative phase in increments of 0.1*π* radians from 0.1*π* to 1.9*π* radians.

Fig. 12 shows the magnitude of the frequency content at 2 kHz of each recording for the HyperSound speakers canted in at 4 degrees, with the gain of the speakers set equal to each other. By increasing the beam interaction we again found that there was a more pronounced length limited effect.

**Fig. 13:**
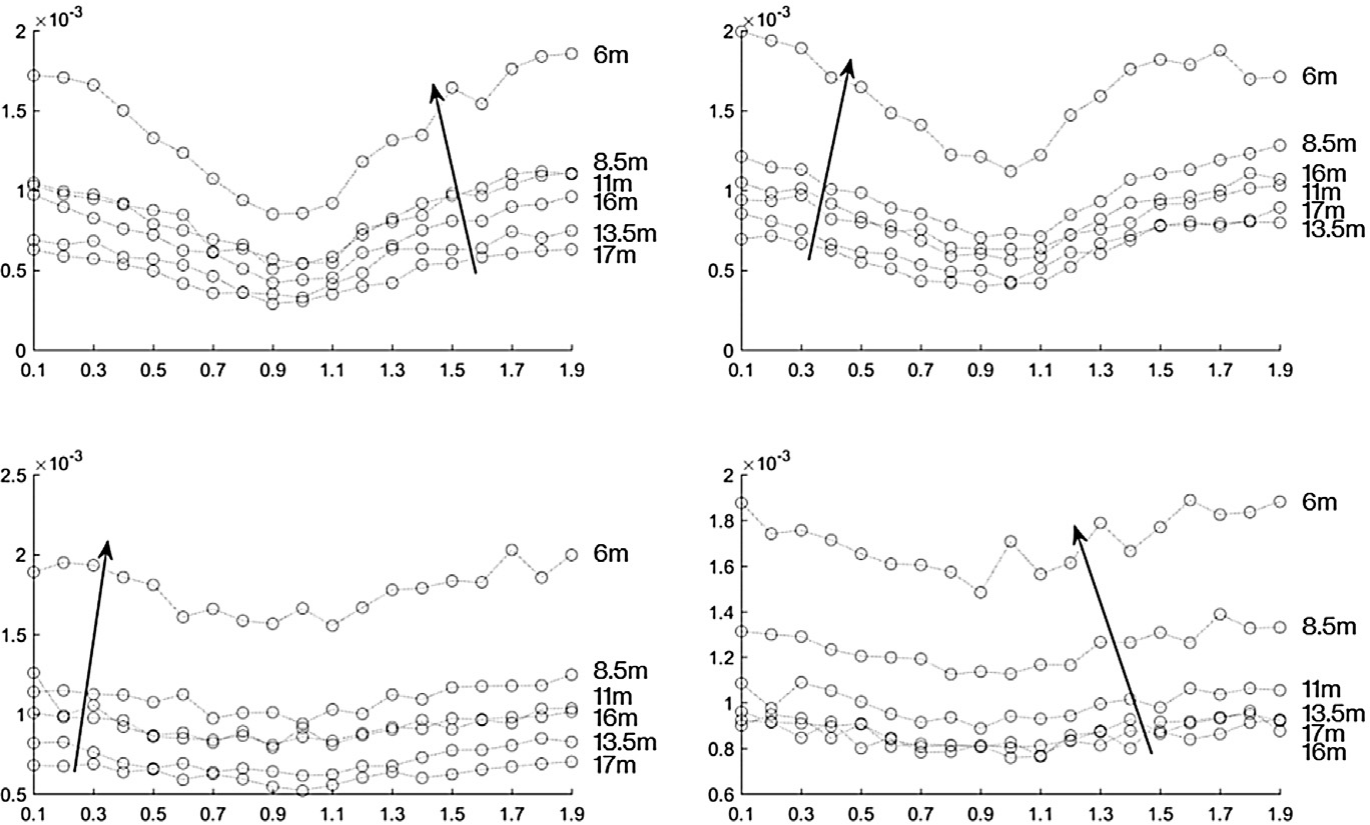
The magnitude of the recorded 2 kHz signal versus phase difference at various distances for the HyperSound speakers canted in at 4 degrees. The top plots show the measurements with a gain offset of 10 dB. The bottom plots show the measurements with a gain offset of 20 dB. The plots on the right are when speaker one had the lower gain and the left side is when speaker two had the lower gain.

Fig. 13 shows the magnitude of the frequency content at 2 kHz of each recording for the HyperSound speakers canted in at 4 degrees. THe top plots show when one speaker had a gain of −10 dB and the other speaker had a gain set at 0. The plot on the right side shows when speaker one had the gain set at −10 dB and in the left plot speaker two had the gain set at −10 dB. In these plots the length limited effect is apparent, especially in the measurement taken closest to the speakers. The bottom plots of Fig. 13 also shows the magnitude of the frequency content at 2 kHz of each recording for the HyperSound speakers canted in at 4 degrees, when one speaker had a gain of −20 dB and the other speaker had a gain set at 0. The plot on the right side shows when speaker one had the gain set at −20 dB and in the left plot speaker two had the gain set at −20 dB. It can be seen that having the gain of one speaker at −20 dB had no significant length limited effect in comparison to that of the speakers at the same volume.

## 4 Discussion

This experimental study shows that the length limited effect is a measurable phenomenon that can be produced using commercial off the shelf parametric array speakers in the open air. By taking advantage of the signal processing in the commercial parametric amplifiers we propagated pairs of 2 kHz narrow sound beams with parametric array speakers. We generated the length-limiting effect using pairs of parametric arrays mounted so that the directional audio beams were simultaneously co-propagating through the air, with their relative phase adjusted for varying amount of interference between the two sound beams. We believe that the length limited effect is created from the constructive and destructive interference in the audible signal created by the parametric arrays. We have shown that the length limited effect can be produced without strict control of the ultrasound primary signals.

Increasing the interaction volume between the audible sound beams by canting the speakers slightly inward increased the effectiveness of the length limited phenomenon for the AudioSpotlights because their 24-inch by 24-inch size gives a highly directional beam. When positioned adjacent to each other and aimed in parallel, the beams did not overlap sufficiently, but canting them inwards 4 degrees did give enough beam overlap. When the AudioSpotlights were canted inward at 10 degrees, however, the two beams did not interact sufficiently to produce a length-limited effect. For the smaller, rectangular HyperSound speakers there was enough beam spread that even when they were pointed in parallel, the beams interacted to give a length-limited effect.

We found that keeping the relative magnitude of the sound projected through the speakers the same produced the strongest length limited effect. Changing the gain of one of the speakers did not have significant impact on the length limited effect. And adjusting the gain of one speaker to −20 dB in some cases eliminated the length limited effect entirely.

The results presented here are a step towards understanding and ultimately harnessing length-limited sound beams for practical applications. Current work underway in our laboratory involves 3D non-linear acoustic simulations to begin to optimize the method.

## Footnotes

1 https://www.zoom-na.com/products/field-video-recording/field-recording/h2-handy-recorder.

2 https://web.archive.org/web/20071011022341/http://www.zoom.co.jp/english/products/h2/.
